# Nanosomes in Precision Nanomedicine

**DOI:** 10.3390/nano14211717

**Published:** 2024-10-27

**Authors:** Lucia Baldino

**Affiliations:** Department of Industrial Engineering, University of Salerno, via Giovanni Paolo II, 132, 84084 Fisciano, Italy; lbaldino@unisa.it

Nanosomes are vesicles that can be used in precision nanomedicine to deliver active pharmaceutical ingredients to specific cells or tissues; they are designed to improve the efficacy and safety of drug delivery systems. Nanosomes, like liposomes, niosomes, transfersomes, etc., are characterized by a spherical morphology whose structure consists of a lipidic bilayer around a hydrophilic core. The core generally contains a therapeutic drug, whereas the external surface can be coated by functional biomolecules that can selectively target specific cells or tissues. The use of nanosomes in precision nanomedicine has the potential to revolutionize the treatment of various diseases, including cancer, neurological disorders, and infectious diseases, simultaneously reducing the side effects associated with traditional drug delivery systems. Furthermore, nanosomes can be modified to carry multiple therapeutic drugs, allowing for personalized medicine tailored to individual patient needs.

In this context, Baldino et al. [1] produced niosomes using a supercritical CO2-assisted process operated at 100 bar and 40 °C. The formulation containing cholesterol and 80:20 Span 80/Tween 80 was selected to encapsulate vancomycin; a drug release study was carried out to highlight the effect of PEGylation. The results showed that nanometric vesicles characterized by a high drug encapsulation efficiency (95% for non-PEGylated and 98% for PEGylated niosomes) were obtained. Moreover, PEGylated niosomes prolonged the vancomycin release time by up to 20-fold with respect to untreated drug powder. Pan et al. [2] synthesized an MOF-derived Fe-N-C nanozyme to create biosensors for the coulometric and visual detection of alkaline phosphatase (ALP). In particular, they found that Fe-N-C nanozymes can efficiently oxidize 3,3′,5,5′-tetramethylbenzidine (TMB) to generate blue-colored tetramethyl benzidine (TMBox) without the need for H_2_O_2_. They also developed a visual detection tool for ALP by using a smartphone-based assay, as well as facilitating practical and accessible point-and-care testing (POCT) in resource-limited areas. Squittieri et al. [3] proposed transfersomes as deformable vesicles that can transport drugs across difficult-to-permeate barriers in human tissues. Specifically, they produced nano-transfersomes using a supercritical CO_2_-assisted process. Formulations prepared using an 80:20 weight ratio of Span^®^ 80 and phosphatidylcholine produced stable transfersomes (−30.4 ± 2.4 mV ζ-potential) that were characterized by a mean diameter of 138 ± 55 nm. A prolonged ascorbic acid release was also recorded when the largest amount of phosphatidylcholine (3000 mg) was used. In their review, López-Goerne et al. [4] highlighted that catalytic nanomedicine achieved important advances in developing bionanocatalysts and brain-tissue-biocompatible catalytic nanostructures that are capable of destabilizing the genetic material of malignant cells, causing their apoptosis. Li et al. [5] proposed the incorporation of doxorubicin (DOX) and anti-angiogenesis agent combretastatin A4 (CA4) into poly(lactic-co-glycolic acid) (PLGA)-based co-delivery nanohybrids (PLGA/DC NPs) via an improved double emulsion technology. After that, polydopamine (PDA) was modified on the PLGA/DC NP surface through the self-assembly method for photothermal therapy. Both in vitro and in vivo studies demonstrated that PDA@PLGA/DC NPs enhanced cytotoxicity under laser irradiation, and combined therapeutic effects were obtained when DOX, CA4, and PDA were integrated into a single nanoplatform. Nefedova et al. [6] used metallic silver nanoparticles (AgNPs) to address the global problem of antibiotic resistance. In vivo fieldwork was carried out with 200 breeding cows with serous mastitis. Ex vivo analyses showed that after the cow was treated with an antibiotic-containing drug DienomastTM, E. coli sensibility to 31 antibiotics decreased by 27.3%, but after treatment with AgNPs, it increased by 21.2%. This could be explained by the 8.9% increase in the portion of isolates showing an efflux effect after DienomastTM treatment, while treatment with Argovit-CTM resulted in a 16.0% drop. Zhang et al. [7] designed a reactive oxygen species (ROS)-cleavable nanoparticle system (MXene-TK-DOX@PDA) for chemotherapy drug delivery and antibacterial applications. These nanoparticles demonstrated an outstanding photothermal conversion efficiency, a superior photothermal stability, and a remarkable extinction coefficient (23.3 L g^−1^ cm^−1^ at 808 nm). In addition, MXene-TK-DOX@PDA nanoparticles showed a high antibacterial activity against both Gram-negative and Gram-positive bacteria. Menichetti et al. [8] reviewed the application of polydopamine (PDA), which is one of the most interesting materials in nanomedicine because of its versatility and biocompatibility. This polymer can be functionalized to favor cellular uptake and blood circulation, and it can also induce drug release through NIR light irradiation and pH. Qi et al. [9] summarized the research progress of three types of composite nanomaterials, including organic composite materials, inorganic materials, and organic–inorganic hybrid materials, used as antibiofilms with non-phototherapy and phototherapy modes of action, with the aim of proposing the design of safe and efficient antibiofilm materials.

This overview demonstrates that the research field of nanomedicine is rapidly and widely expanding and following different approaches thanks to the interest and the collaboration among scientists with a heterogeneous background. The development and application of innovative organic and inorganic biomaterials in the nanotechnology sector favors this growing process, with a specific attention to the sustainability that accompanies all the production steps up to the treatment of patients. The principle aim is reaching a personalized cure with limited side effects that can be used worldwide.
